# Hybrid ICA-Seed-Based Methods for fMRI Functional Connectivity Assessment: A Feasibility Study

**DOI:** 10.1155/2010/868976

**Published:** 2010-06-28

**Authors:** Robert E. Kelly, Zhishun Wang, George S. Alexopoulos, Faith M. Gunning, Christopher F. Murphy, Sarah Shizuko Morimoto, Dora Kanellopoulos, Zhiru Jia, Kelvin O. Lim, Matthew J. Hoptman

**Affiliations:** ^1^Weill Cornell Institute of Geriatric Psychiatry, Weill Cornell Medical College, 21 Bloomingdale Road, White Plains, NY 10605, USA; ^2^The MRI Unit and The Division of Child and Adolescent Psychiatry, Columbia University, New York State Psychiatric Institute (NYSPI), 1051 Riverside Drive, New York, NY 10032, USA; ^3^Department of Psychiatry, University of Minnesota, 717 Delaware Street SE, Minneapolis, MN 55414, USA; ^4^Division of Clinical Research, Nathan S. Kline Institute for Psychiatric Research, 140 Old Orangeburg Road, Orangeburg, NY 10962, USA; ^5^Department of Psychiatry, New York University School of Medicine, 550 First Avenue, New York, NY 10016, USA

## Abstract

Brain functional connectivity (FC) is often assessed from fMRI data using seed-based methods, such as those of detecting temporal correlation between a predefined region (seed) and all other regions in the brain; or using multivariate methods, such as independent component analysis (ICA). ICA is a useful data-driven tool, but reproducibility issues complicate group inferences based on FC maps derived with ICA. These reproducibility issues can be circumvented with hybrid methods that use information from ICA-derived spatial maps as seeds to produce seed-based FC maps. We report results from five experiments to demonstrate the potential advantages of hybrid ICA-seed-based FC methods, comparing results from regressing fMRI data against task-related *a priori* time courses, with “back-reconstruction” from a group ICA, and with five hybrid ICA-seed-based FC methods: ROI-based with (1) single-voxel, (2) few-voxel, and (3) many-voxel seed; and dual-regression-based with (4) single ICA map and (5) multiple ICA map seed.

## 1. Introduction

Functional connectivity (FC), broadly defined, is “correlations between remote neurophysiological events” [1]. There are a number of approaches for the detection of FC from blood-oxygen level dependent (BOLD) signals measured by functional magnetic resonance imaging (fMRI) of the brain. A commonly implemented approach is a “seed-based” approach that can be applied with a general linear model (GLM) using time course regressors derived from selected brain regions to find other brain regions having correlated BOLD signal activity patterns [2, 3]. Another commonly used data-driven approach is independent component analysis (ICA), which derives spatiotemporal components (pairs of spatial maps and associated time courses) through blind signal source separation and linear decomposition of fMRI data [4, 5]. Independent component (IC) spatial maps represent constellations of brain regions that are partially synchronized with the corresponding component time courses. ICA in this article is synonymous with spatial ICA, the most common implementation of ICA used with fMRI data, which allows components to be temporally correlated, but restricts them to be spatially independent.

Both the seed-based approach and ICA have produced similar FC maps in experiments where no experimentally-derived time course was available (e.g., resting-state) [6–8]. Both approaches have produced pairs of spatial maps and associated time courses comparable to those produced by standard GLM techniques that regress fMRI data against experimentally derived *a priori *hemodynamic response functions, in experiments driven by task activities with known time courses [9–17]. The results derived with ICA are sometimes more consistent with expected BOLD response patterns than those derived with the *a priori*, task-related approach, especially for transient task-related BOLD responses or responses whose time courses are difficult to predict beforehand [4, 18–21]. ICA does not require an *a priori* time course or choice of seed region, making it ideal for exploratory studies. In some cases, ICA can be more sensitive, specific, and accurate in showing functionally connected regions than seed-based approaches [22, 23], as we demonstrate with an example (Experiment 1).

Despite potential advantages of ICA over task-related or seed-based GLM approaches in visualizing correlated brain activity, ICA reproducibility issues may complicate or limit ICA's use in the generation of FC maps to be used for group comparison. Temporally correlated ICs can sometimes be expressed as a single component [4, 24, 25], and vice versa [26]. Iterative ICA algorithms that use a random seed can produce different sets of components on separate analyses performed on exactly the same data [27, 28]. From one ICA to the next such components can appear to be modified, split into two components, or to “disappear” completely by having their variance redistributed across multiple components [29]. Such variations in ICs also occur during “bootstrapping,” where individual ICAs are performed on separate portions of a dataset involving similar tasks [27, 28], and when analyzing separate subjects performing the same tasks [16]. ICA's variability in parsing fMRI data variance into separate ICs can complicate comparison of an IC spatial map generated from one subject's (or session's) data to similar IC spatial maps generated from other subjects' (or sessions') data [30], because the signal sources represented by each IC in any group of relatively homologous ICs (ICs with closely matching spatial patterns across subjects/sessions) may not exactly match the signal sources represented by the other ICs in the group. Addressing these ICA reproducibility issues is a subject of ongoing research [12, 16, 31, 32].

Some of these reproducibility issues may be avoided by comparing FC spatial maps generated through seed-based methods. Such methods usually select seed brain regions based on information derived from theoretical considerations [33], task-related GLM studies [34–36], or ICA studies [10]. Deriving seed information from ICA can be considered a hybrid ICA-seed-based approach where the exploratory power of ICA is utilized to select a seed from a huge number of possible choices. Literature on the subject of the potential benefits and limitations of such an approach is scarce. However, other ICA-based approaches for addressing these reproducibility issues have been described [12, 26, 31, 37, 38]. Two of the most widely implemented of these approaches that can be applied in a data-driven fashion, without *a priori* time courses, or any other prior knowledge from an experimental paradigm, are the “back-reconstruction” [12, 37] and “dual-regression” [31, 39] methods. Both methods use data from a group ICA performed on the temporally concatenated fMRI datasets of all subjects/sessions in a group as the source of data to derive individual subject/session spatial maps that can then be compared to draw inferences about individual or subgroup differences. Here, we report the results of comparing five hybrid ICA-seed-based methods to each other and to these two methods, using standards of comparison derived from knowledge of visuomotor experimental paradigms involved during collection of fMRI data, and from results of fMRI analyses with task-related GLM methods (i.e., GLM applied using time course regressors derived from the experimental paradigms).

## 2. Theory

The GLM is often expressed in matrix notation as [3, 20, 40–43]

(1) X=Gβ+ϵ,

where **X** is a *t* × *v* matrix (of fMRI data after preprocessing) containing one row for each point in time (volume acquisition) and one column for each point in space (voxel). Each row represents the spatial map (BOLD signal at each voxel) of a volume of data (*t* = number of volumes), and each column represents the time course for a single voxel (*v* = number of voxels). **G** is a *t* × *s* design matrix where each column contains the hypothesized time course of the BOLD response for a signal of interest (*s* = number of signals of interest) or for a covariate of no interest. **β** is an *s* × *v* matrix where each row contains a spatial map of parameters to be estimated (one parameter for each voxel) corresponding to one of the time courses (the *i*th row of **β** corresponds to the *i*th column of **G**). **ϵ** is a *t* × *v* matrix of residual errors. The parameters in **β** can be estimated through standard, least-square linear regression techniques, minimizing the sums (across all time points) of the squared residual error terms for each voxel. These maps of parameter estimates (with other information, such as the parameter estimate standard errors) can be used for hypothesis testing of each time course of **G** in various regions of the brain. They can also provide the basis for group comparison statistics, derived from comparing the spatial maps for different conditions or different groups of subjects.

The spatial maps of **β** in (1) are uniquely determined by least-square linear regression if the time courses in the design matrix **G** are completely specified and none of the time courses are colinear. Similarly, the time courses in **G** can be uniquely determined through GLM by regressing the fMRI data against completely specified, non-colinear spatial maps in **β**. This relationship is readily apparent if we convert (1) to an equivalent form by transposing matrices on both sides of the equation and apply the identities (**A** + **B**)^**T**^ = **A** ^**T**^ + **B** ^**T**^ and (**A** **B**)^**T**^ = **B** ^**T**^ **A** ^**T**^ to yield

(2) $$X^{T}=\beta^{T}G^{T}+\epsilon^{T}.$$

Least-square linear regression to determine the time courses in **G** proceeds in the same manner as for determination of the spatial maps of **β** in (1), except that the sums (across all *voxels*) of the squared residual error terms for each *time point* are minimized to determine **G**.

ICA, like GLM, is based on a linear model. For a general case, one may consider including an error term in a standard ICA model, like the model used in probabilistic independent component analysis (PICA) [11]. In this study, we use this form of ICA model, where the relationships among the preprocessed fMRI data, spatial maps, and time courses can be expressed as

(3) X=MC+E.

**X** here is defined in the same manner as for (1) and represents the same data, while **M, C, **and** E** are matrices that represent time courses, spatial maps, and error terms in the same format as for **G**, **β**, and **ϵ**, but that generally do not contain the same matrix values, and the number of time courses and associated spatial maps in **M** and **C** may differ from the number in **G** and **β**. The entries in **E** are error terms that represent predominately random, Gaussian noise. They are derived using principle component analysis (PCA) with dimensionality reduction to separate the data into **E** and a portion that represents predominately neural signals and structured, nonrandom noise (**MC)**. ICA then solves for both **M** and **C** simultaneously using only the fMRI data (minus **E**) and some assumptions concerning the form that the solution should take (e.g., nonrandom components whose spatial maps are statistically independent).

In this article we broadly define seed-based FC as the result of a two-step process. The first step derives one or more time courses from one or more “seeds” (spatial maps or portions thereof) and the fMRI data being analyzed. The second step regresses (GLM) the fMRI data against the time courses to derive corresponding FC spatial maps. A simple case of seed-based FC is region-of-interest-based (ROI-based) FC derived by averaging the fMRI data time courses of each voxel within a predefined ROI (the “seed”) to produce a time course against which the fMRI data is regressed to yield a single FC spatial map. A more complex case of seed-based FC involves dual regression, a process where fMRI data is regressed against a set of predefined spatial maps to derive a set of time courses against which the fMRI data is regressed once more to produce a corresponding set of FC spatial maps [31]. If the predefined spatial maps consist of some spatial maps of interest and some nuisance covariate maps of no interest, then the corresponding time courses and FC maps can also be divided into those of interest and those of no interest.

## 3. Materials and Methods

### 3.1. Overview

Five experiments were performed to evaluate the performance of the five hybrid ICA-seed-based FC methods. Experiment 1 illustrated the potential advantages of a hybrid ICA-seed-based method over a seed-based method that does not utilize information from ICA, using natural data from an fMRI run, with artificial data added. Experiment 2 tested the performance of the five hybrid methods, comparing results to those obtained from task-related GLM, using six runs of data from a single participant. Experiment 3 tested the performance of the five hybrid methods, comparing results to those obtained from task-related GLM and two methods for extracting information about individual subjects, based on group ICA. The data source was an fMRI run from each of 14 participants. Finally, in Experiments 4 and 5 the effects that ICA dimensionality might have on the hybrid methods were evaluated by repeating Experiment 3 using a smaller (5) and larger (30) number of ICs (instead of 14).

### 3.2. Participants

#### 3.2.1. Experiments 1 and 2

A right-handed adult male volunteer was recruited after informed consent, from among students in an fMRI lab course at Columbia University. Handedness was determined by the laterality quotient (+100) from the Edinburgh Handedness Inventory [44, 45]. The study was approved by an Institutional Review Board at Columbia University.

#### 3.2.2. Experiments 3–5

14 healthy, right-handed adults were recruited in accordance with institutional guidelines approved by the Medical College of Wisconsin and the University of New Mexico School of Medicine.

### 3.3. Experimental Paradigms

#### 3.3.1. Experiments 1 and 2

fMRI data were collected from an experiment originally intended as a pilot study of attachment theory [46–48]. The participant provided digitized (electronic) photos of a male friend, a female friend, photos of his parents taken during his childhood, and recent parental photos. To these six photos were added a photo of a famous person similar in appearance to each of the young parental images and a photo of a complete stranger similar to each of the older parental photos. The participant was briefly shown the images in advance of the experiment to ensure recognition of the famous faces and lack of recognition of the strangers.

The photo images were processed using Adobe Photoshop Elements (version 2.0) to make them as similar as possible in appearance apart from the forms of the faces. Background scenes were eliminated and replaced with a dark grey background, making a smooth transition in brightness toward the edges of the face image to avoid sharp boundaries at the periphery of the faces. Photos were converted from color to grayscale and adjusted for brightness, contrast, head size, and blurriness of image (making sharper photos grainier and blurrier). The facial images were cropped from the chin up, leaving enough room at the top to ensure that the bridges of the nose were vertically at the center of each image. The width of each image was cropped to whatever length was necessary to show the face from ear to ear. Each photo was pixelized to 72 dpi with a height of 400 pixels.

The participant provided the names of each person in the photos. Strangers were represented by the words “a woman” or “a man.” Word images of the names were generated by Presentation software (version 10.2), adjusted in size so that the longest names (Rita Hayworth and Frank Sinatra) took up half of the full width of the image display, the same as for the widest photos. The heights of capital letters were approximately 10 times smaller vertically than the photos. The word images were colored either blue or green and shown on a medium-gray background. The photos were shown on the same background and colored either blue-gray or green-gray using 30% opacity.

**Figure 1:**
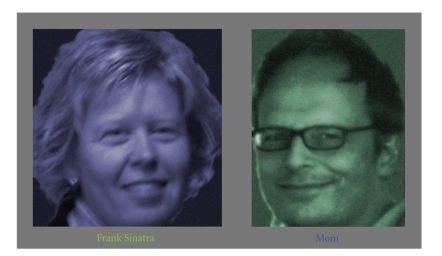
Examples of photos and names displayed during fMRI data collection.

The 10 photos and names in two different colors yielded a total of 40 images (Figure 1), which were each shown in pseudorandomized order exactly once during each of the six runs. The pictures were each presented for an average of 9.0 seconds (random durations of 7.5–10.5 seconds), with a blank, medium-gray screen shown between images for 0.5 seconds, yielding an average run time of 380 seconds. The subject was given about 1 minute rest in between runs. He was given ear plugs, had his forehead taped down, and was equipped with a button-box while in the scanner.

In order to maximize attention and brain activity and minimize sources of data noise, the subject was instructed to be as still as possible (avoiding swallowing) during fMRI runs, focusing eyes on the center of the screen at all times (trying to avoid blinking during image presentation), and to press one button for blue images and another for green images as soon as he was sure of the color of the image. He was asked to concentrate as continuously and exclusively as possible on the person corresponding to each image as it was being shown, and to imagine that he was greeting the person in a usual setting where he might see the individual (e.g., for famous people, on television).

#### 3.3.2. Experiments 3–5

fMRI data were used from a study of the association between brain hemispheric lateralization patterns and motor task complexity [49]. The publicly available data was downloaded from The fMRI Data Center (http://www.fmridc.org/, accession number 2-2003-114E5). Eight runs were collected in that study, with each run involving either a complex or a simple motor task, and either the left or right hand; two runs were collected for each of these four cases. Each run involved 10, 24-second-long blocks. Each block involved 12 seconds rest followed by 4, 3-second trials where the participants were shown sequences of five digits (1, 2, or 3) and responded by pressing buttons corresponding to the sequence shown (1 = index finger, 2 = middle finger, and 3 = ring finger). The complex condition consisted of heterogeneous sequences, which always used three fingers and four transitions, whereas the simple condition involved repetition of the same digit in each trial. The digits were displayed for 2.5 seconds, and approximately 2 seconds were required for participants to manually enter the digits.

We only used data from the first run of the complex condition performed with the right hand, for the following reasons. First, we chose only one run in order to minimize memory requirements on our 32-bit architecture computers. Second, we chose the complex condition rather than the simple condition because the complex condition involved use of each of the three fingers in every trial, thereby providing a more even use of the corresponding brain regions during the latter half of each block. Third, we chose the first run rather than the second run, because we reasoned that the effects of fatigue would probably make the brain's response to the task weaker during the second run, but this choice was somewhat arbitrary. Finally, we chose the right hand rather than left, because a quick scan and processing of the data with ICA and GLM for the first two subjects revealed that the activation in response to use of the right hand appeared to be stronger than in response to use of the left hand.

### 3.4. Data Acquisition

#### 3.4.1. Experiments 1 and 2

Images were acquired using a 1.5T General Electric (GE) TwinSpeed MRI scanner. Functional scans were performed using EPI-BOLD (TR = 2000 ms; TE = 38 ms; flip angle = 90 degrees; FOV = 19.2 cm; 64 × 64 matrix; 29 oblique-axial slices 4.5 mm thick; skip 0 mm; interleaved acquisition; voxel size = 3 × 3 × 4.5 mm). Immediately after the functional scans, a 10-minute, high resolution, T1-weighted structural MRI image was acquired using the 3D SPGR sequence (186 slices; 256 × 256; FOV = 256 mm; voxel size = 1 × 1 × 1 mm).

#### 3.4.2. Experiments 3–5

Images were acquired using a 1.5-T GE Signa scanner. Echo-planar (EP) images were collected using a single-shot, blipped, gradient-echo EP pulse sequence (TR = 4000 ms; TE = 40 msec; FOV = 24 cm; 64 × 64 matrix; 22 contiguous sagittal 6-mm thick slices; voxel size = 3.75 × 3.75 × 6 mm). Prior to functional imaging, high-resolution 3D spoiled gradient-recalled at steady-state anatomic images were collected: TR = 24 msec, TE = 5 msec, flip angle = 40 degrees, number of excitations = 1, slice thickness = 1.2 mm, FOV = 24 cm, and resolution = 256 × 128.

### 3.5. Image Preprocessing

fMRI image preprocessing for all experiments was carried out using FSL (version 4.1), FMRIB's Software Library (http://www.fmrib.ox.ac.uk/fsl) [50, 51], involving nonbrain removal using BET [52]; motion correction with MCFLIRT [53]; slice-timing correction for interleaved acquisitions using Fourier-space time-series phase-shifting; highpass temporal filtering using Gaussian-weighted least-squares straight line fitting (with *σ* = 27.5 seconds for Experiments 1 and 2, and *σ* = 50 seconds for Experiments 3–5); spatial smoothing using a Gaussian kernel with full-width half-maximum 8 mm; coregistration to high-resolution T1-weighted images; and normalization of all images to standard space (MNI, Montreal Neurological Institute atlas, using resolutions of 3 × 3 × 3 mm for Experiments 1 and 2, and 4 × 4 × 4 mm for Experiments 3–5, which were the smallest voxel sizes that did not result in memory overflow errors while performing group ICAs) with affine registration, FSL FLIRT [53, 54].

### 3.6. ICA Processing

#### 3.6.1. Experiments 1 and 2

ICAs were performed using PICA [11] as implemented in FSL's MELODIC (Multivariate Exploratory Linear Decomposition into Independent Components) version 3.09, which can be considered a variant of FastICA [55] where components are estimated simultaneously rather than one at a time. This process involved masking of nonbrain voxels, voxelwise demeaning of the data, normalization of the voxel-wise variance, whitening, projection into an *N*-dimensional subspace using PCA, and decomposition into *N* time courses and corresponding spatial maps (spatiotemporal ICA components) by optimizing for non-Gaussian spatial source distributions using a fixed-point iteration technique [55]. *N* was estimated using the FSL default, Laplace approximation [11, 56]. Estimated component maps were divided by the standard deviation of the residual noise and thresholded by fitting a mixture model to the histogram of intensity values. For Experiment 1, IC spatial maps were thresholded conservatively, using a mixture model and alternative hypothesis testing approach, with the “mmthresh” parameter set to 0.95. For Experiment 2, the FSL default value of mmthresh = 0.5 was used.

ICs produced with PICA can vary, even if repeated with identical fMRI data, because PICA is an iterative approach based on a random seed [11]. For this reason, in Experiments 1–3 we repeated PICA 10 times every time it was used, so that we could arrive at a best estimate by averaging the 10 sets of component spatial maps and time courses in the series [37]. However, we found that for Experiments 1–3, in all 10 cases PICA produced component spatial maps and time courses that from visual inspection appeared to be identical. An examination of the exact time course values revealed that in most cases they were identical, and when they were not, in all cases they differed from the first PICA results in the series by less than 3 × 10^−5^ (for time courses normalized to zero mean and unit variance), a figure comparable to rounding error. For this reason, rather than averaging, we used the results from the first PICA in each series of 10. The reason we encountered negligible differences among members of each series of 10 may have been that we chose a very small value (10^−8^) of “epsilon,” the minimum error change parameter used to determine at what point the ICA algorithm has converged on a single solution. At this setting of epsilon, convergence generally required relatively few steps (less than 300), and allowing many steps did not help in cases of nonconvergence; so we used the default maximum number of iterations before restart (500).

#### 3.6.2. Experiments 3–5

Group ICA was performed with MELODIC, using parameters as for Experiments 1 and 2, with the multisubject temporal concatenation option, which performed PICA on the normalized data from all 14 participants, temporally concatenated into a single group fMRI “run.” Some or all of the group ICs were the seed source for the five hybrid ICA-seed-based approaches. In addition, a group ICA was performed using the Group ICA of FMRI Toolbox (GIFT, v1.3 g) implemented in Matlab (http://icatb.sourceforge.net/), using two data-reduction steps. This group ICA approach, described in [12], like PICA began with subjectwise, temporal concatenation of the fMRI data, followed by whitening and projection into an *N*-dimensional subspace using PCA. *N* was specified to be the same as the number of components that were estimated for the PICA group ICA (derived using the Laplace method). Group ICs were then generated using Infomax (the GIFT default), a neural network algorithm that attempts to minimize the mutual information of the network outputs [12, 30, 57]. Finally, individual-subject spatial maps and time courses corresponding to each group IC were derived using the “regular” back-reconstruction method [12]. Z-score scaling was applied to the group and individual-subject spatial maps, normalizing them to zero mean and unit variance. These maps were thresholded for figure display at *z* > 3.1 (one-tailed *P* < .001) as in [12, 58].

### 3.7. GLM Processing

Individual-subject spatial maps not derived directly from ICA were derived with GLM by regressing fMRI data against one or more time courses using FSL FEAT with FILM local autocorrelation correction [59] to generate Gaussianised, *z*-statistic images reflecting parameter estimates in relation to their standard errors and degrees of freedom. The time course for each task-related GLM analysis was generated by convolving an experimentally derived time course against a Gaussian function with peak lag = 5 seconds and *σ* = 2.8 seconds. Time courses derived from seed-based approaches were not modified. For Experiments 1, 3, 4, and 5, z-statistic images were processed by maximum-height thresholding voxels based on Gaussian Random Field Theory (GRFT) to a corrected, one-tailed significance of *P* ≤ .05 [60]. For Experiment 2, *z*-statistic images were thresholded by cluster size determined by counting contiguous (26-neighbor) voxels with *P* < .01 (one-tailed, *z* > 2.3) and applying GRFT to determine a corrected cluster size threshold corresponding to one-tailed significance of *P* ≤ .05 [60]. We chose the more conservative voxel-thresholding approach for Experiments 1, 3, 4, and 5 because we judged that tightly controlling false positives would be more suitable for the visual-inspection-based comparisons of Experiment 1 and the assessments of receiver operating characteristics of Experiments 3–5. We chose the more sensitive cluster-based thresholding for Experiment 2 in order to better visualize “activation” in the right temporal occipital fusiform cortex (rTOF), where the “fusiform face area” (FFA) is located, a region commonly involved during experimental tasks with imagined or perceived images of faces [61–63]. Such “activation” was present in the rTOF for task-related GLM analyses in 5/6 runs with cluster-based thresholding, compared with only 3/6 runs with voxel-based thresholding.

### 3.8. Experiment 1

**Figure 2:**
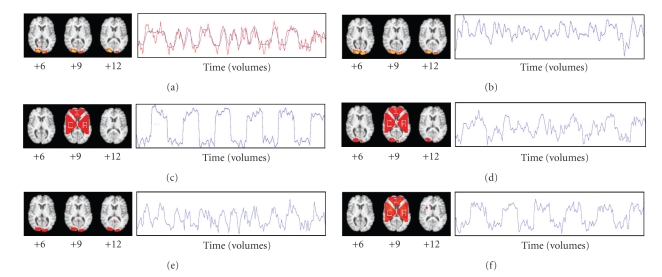
Spatial maps and time courses derived from task-related GLM, ICA, seed-based approach, and hybrid ICA-seed-based approach. Numbers below axial brain slices indicate *z*-coordinate (MNI). Blue lines: time courses from linear model. Red lines: fMRI data deviating from model, at voxel with best fit to model. (a) Task-related GLM. (b) Natural IC. (c) Artificial IC. (d) Seed-based FC. (e) Hybrid FC, seed from natural IC. (f) Hybrid FC, seed from artificial IC. The bright yellow dots (d–f) show seed locations.

Task-related GLM was applied to the preprocessed fMRI data from Run 1 (Figure 2(a)), and the location (MNI coordinates 21, −93, 9) of the voxel with the highest z-score (10.6) was determined. Artificial signal was added to the axial slice containing that voxel, creating an artificial component whose variance was comparable to the variance of the natural occipital component (Figure 2(b)). The artificial signal was added to the voxel at coordinates 21, −93, 9 in addition to all voxels in three large triangular regions outside of the occipital cortex (Figure 2(c)). The artificial signal consisted of a square wave with a period of 60 seconds (30 volumes) and amplitude at each voxel corresponding to ±0.5% of the mean MR signal intensity for the voxel over time. Adding the artificial signal before fMRI preprocessing would have better simulated neural signal, but we added it after preprocessing to sharpen image boundaries for the illustrative purposes of this experiment. Both components were visualized with ICA (Figures 2(b) and 2(c)).

The voxel at coordinates 21, −93, 9 was chosen as the seed to produce an ROI-based FC map containing both the natural and artificial components (Figure 2(d)). We repeated this seed-based FC analysis twice more (Figures 2(e) and 2(f)), choosing the voxel in the *z* = 9 slice with the highest z-score from the natural (coordinates 12, −99, 9) and artificial (coordinates 30, 9, 9) IC spatial maps. Results were compared with visual inspection, and statistics were gathered on sensitivity and specificity for correctly identifying voxels where artificial signal had been added.

### 3.9. Experiment 2

**Figure 3:**
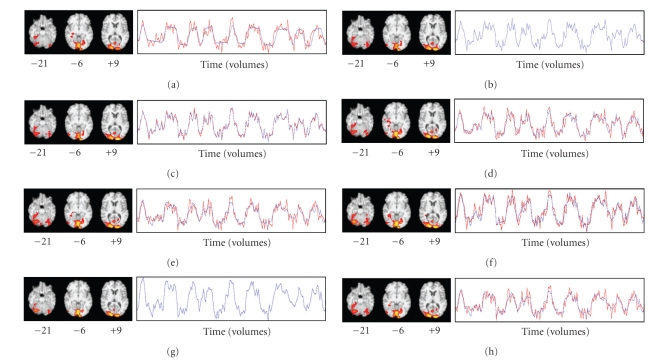
Spatial maps and time courses for Run 3, derived from (a) task-related GLM; hybrid ICA-seed-based approaches using the average time course from (b) a single voxel, (c) a few voxels, or (d) many voxels; dual regression using seed spatial information from (e) an IC spatial map or (f) an entire set of IC spatial maps; (g) ICA; and (h) regression of the fMRI data against the IC time course. Run 3 was the source of seed spatial information.

We compared the results of task-based GLM to the results of five hybrid ICA-seed-based FC methods using preprocessed fMRI data without added artificial signal. The seed source for each method was the collection of IC spatial maps (13 in all) derived from ICA of Run 3's fMRI data. The spatial map and time course of the last (ranked in descending order of explained variance) of these components (IC13) resembled the spatial map and time course derived from task-related GLM (Figure 3(a)). Run 3 was chosen because it was the run whose task-related GLM analysis had the highest z-score (11.9) in any voxel: we hypothesized that Run 3 might therefore be most representative of the activation pattern we were studying.

The first hybrid method (single-voxel seed, SV) used the voxel (coordinates −12, −102, 3) in IC13 with the highest z-score (18.27) as the seed for ROI-based FC. Thus, FC maps were derived for each of the five target runs (i.e., nonseed Runs 1, 2, 4, 5, and 6) by regressing their fMRI data against their seed voxel time course (i.e., the time course of the voxel at coordinates −12, −102, 3 for the run being analyzed). The second hybrid method (few-voxel seed, FV) derived ROI-based FC using the same procedure, but the regressor for each run was the average time course over a few seed voxels. Those were the voxels (five in all) whose z-score was higher than 17.27, one less than the highest z-score. We thresholded by z-score rather than arbitrarily choosing the number of voxels to be used. The third hybrid method (many-voxel seed, MV) derived ROI-based FC using as the regressor the average time course over many seed voxels, chosen as close to 100 in number as possible by thresholding with whole z-scores. A threshold of *z* > 13 yielded 118 voxels. The fourth hybrid method (dual regression with single-IC seed, DRS) used IC13's spatial map as the seed for dual-regression-based FC. The fMRI data for each of the target runs was regressed against IC13's spatial map using FSL's dual_regression program (beta version 0.3) to yield a time course, against which the fMRI data was regressed to produce an FC map for each run. The fifth hybrid method (dual regression with all ICs as the seed source, DRA) used all 13 ICs as the seed for dual-regression-based FC, using the same procedure as for the fourth method, to yield time courses against which the fMRI data was regressed to produce corresponding FC maps for each target run. The FC map corresponding to IC13 was treated as the FC map of interest.

Each hybrid method was evaluated qualitatively through visual inspection of all spatial maps and time courses generated in this experiment and quantitatively according to the following criteria.

1. *FC map correlation with task-related GLM spatial map from same run > 0*: we did not assume that a successful hybrid method FC map would correlate highly with the task-related GLM map from the same run because the correlations of the GLM maps from the six runs with each other were in the range 0.13–0.45, and differences in latency of hemodynamic responses [64, 65] could potentially result in considerable differences between the FC maps and task-related GLM maps. However, we did expect the spatial maps to be similar in the occipital cortex, the rTOF, and possibly other brain regions; so we expected the maps to be at least somewhat correlated. FSL's fslcc utility was used to calculate the correlation coefficient between the FC spatial map and task-related GLM spatial map for each of the five target runs and the mean correlation was compared to zero with a two-tailed, one-sample *t*-test. All correlation values in this study were Fisher *z*-transformed before statistical testing and calculation of confidence intervals.
2. *FC map correlation with task-related GLM spatial map from same run > FC map correlation with task-related GLM spatial map from seed run*: for hybrid ICA-seed-based methods to be considered successful, the resulting FC maps should be unique to the fMRI data being regressed rather than merely a reflection of the seed source spatial maps or fMRI data. This criterion was a test of this expectation. However, we expected the hybrid FC maps from target runs to correlate with the task-related GLM spatial map from the seed run because the latter was correlated with the task-related GLM spatial maps from target runs (correlation coefficients ranging from 0.25 to 0.44). Nonetheless, we expected the hybrid FC maps to be more strongly correlated with the task-related GLM maps from the same run than from the seed run. We tested this criterion for each hybrid method with a two-tailed, paired two-sample *t*-test, comparing the mean correlation between hybrid FC maps and the task-related GLM spatial map from the corresponding run to the mean correlation between FC maps and the task-related GLM spatial map from the seed run.
3. *FC map correlation with task-related GLM spatial map from same run—a comparison of each method's mean correlation with that of the other four hybrid methods*: depending upon the results of a one-way repeated-measures ANOVA omnibus test of differences in mean correlations (*α* = 0.05), paired *t*-tests (two-tailed) were performed to test for individual differences in means between methods. A Bonferroni correction for multiple comparisons with *α* = 0.05 was applied (significance at *P* < .005 for each of the 10 method pairs compared).
4. *Overlap of thresholded voxels from FC map with task-related GLM spatial map from same subject—a comparison of each method's overlap across runs with the other four methods of deriving individual-subject FC maps*: we examined the percentage of the thresholded voxels in the task-related GLM map that were also thresholded in the corresponding hybrid FC map. Before making this comparison, we eliminated the effects that the thresholding level might have in making this comparison by adjusting the thresholding z-scores of the FC maps so that each map had the same number of thresholded voxels as the task-related GLM map from the same run. This adjustment was equivalent to making the number of false negatives (negative voxels falling within the thresholded task-related GLM map) equal to the number of false positives (positive voxels falling outside of the thresholded task-related GLM map). We then compared the percentage overlap for each method to the other four methods, across the six runs, using the Wilcoxon signed rank test (two-tailed). This test was not sufficiently powered after correction for multiple comparisons, but we included it to provide a qualitative comparison with the results of Experiments 3–5.
5. *Comparable sensitivity to task-related GLM in detecting activation in the rTOF*: the rTOF was the target area for this sensitivity criterion for two reasons. The first reason was that an increase in activity of portions of the rTOF (and some adjacent brain regions), the FFA, has been reported in response to a wide variety of stimuli involving real or imagined images of faces [61–63, 66, 67]. The exact locations of these portions of the rTOF have varied across subjects and have varied depending upon the context of facial presentation (e.g., unfamiliar versus famous faces) [61, 68–70]. We did not know the exact location of the FFA in our data because no FFA localizer scan had been performed, but we expected some activation in the rTOF in response to viewing faces. For this reason we liberally defined our criterion for success to be any thresholded voxels found within the rTOF, defined as voxels whose probability of belonging to the rTOF was ≥0.5 according to the Harvard-Oxford Cortical Structural Atlas (HOCSA) provided with FSL. By this criterion, the task-related GLM method was successful in 4/5 target runs (no activation was found for Run 5, not shown). The low level of “activation” in the rTOF compared with other portions of the occipital cortex was the second reason for targeting the rTOF: we wanted a test that could distinguish task-related GLM from methods with poorer sensitivity. We expected that less sensitive imaging methods would not show any activation. in the rTOF because such activation was barely detectable with task-related GLM. We also reasoned that this test might reveal potential weaknesses with the hybrid approaches because the rTOF was not in close proximity to the regions with the highest z-scores in the GLM- and ICA-derived spatial maps, and therefore the hybrid approaches could not benefit from the effects of smoothing or a tendency for activation patterns to be clustered, making voxel activation patterns similar to those of neighboring voxels. In order to test for statistically significant differences, we compared task-related GLM results with those from each of the hybrid methods using a two-tailed Wilcoxon signed-rank test with *α* = 0.05. Due to the small sample size, the only condition for which a statistically significant difference between task-related GLM and hybrid methods could have been detected would have been the case where a hybrid method did not find rTOF activation in any of the five target runs, and only if we did not correct for multiple comparisons (exploratory study).

### 3.10. Experiment 3

We compared the results of task-based GLM to the results of seven methods of deriving individual-subject FC maps from a group ICA. The group ICs were derived with PICA (FSL) for the first five methods, and with Infomax (GIFT) for the other two. The group IC of interest for each of these methods was selected with visual inspection by comparing group IC spatial maps with the group task-related spatial map (Figure 6(a)) derived from multilevel linear modeling with FSL FLAME (FMRIB's Local Analysis of Mixed Effects) [71, 72] performed on the lower-level, task-related GLM results from all 14 participants (i.e., a “random-effects” analysis). For PICA, the second of 14 ICs ranked by IC variance (PICA IC2, Figure 6(b)) was chosen; for Infomax, the fourteenth (Infomax IC14, Figure 6(c)). The spatial maps of these ICs were similar in appearance and highly correlated (*r* = 0.61).

The first five methods were the same as the hybrid ICA-seed-based methods tested in Experiment 2 (i.e., ROI-based: single-voxel, few-voxels, and many voxels; dual-regression-based: single IC map and all IC maps), using the PICA group ICs as the seed source. For the first method (SV), the seed voxel was located in a region that according to the HOCSA probably corresponded to the left precentral (40% probability) or postcentral gyrus (19%), at MNI coordinates −38, −22, 60. The z-score in PICA IC2 at this point was 13.69. For the second hybrid method (FV), thresholding at *z* = 12.69 yielded 8 more voxels, contiguous with the first voxel. For the third hybrid method (MV), we used the target seed volume from Experiment 2 (100 voxels × 27 *μ*L) rather than the target number of voxels (100) because we did not wish the seed volume to become disproportionately large. Thresholding at *z* = 11 yielded 40 voxels (closest to the target of 42), which were also contiguous. The fourth hybrid method (DRS) used PICA IC2's spatial map as the seed for dual-regression-based FC. The fifth hybrid method (DRA) used all 14 PICA group ICs as the seed for dual-regression-based FC, choosing PICA IC2's spatial map as the FC map of interest. The fifth method as implemented in this experiment is the same as the “multisubject ICA and dual regression” approach described in [31], which can be considered a special case of DRA where the seed ICs are taken from a group ICA performed on the same data being analyzed with the hybrid method. The sixth method (back-reconstruction with Infomax, BR-I) derived the individual-subject ICs using the back-reconstruction method. The seventh method (DRS with Infomax, DRS-I) was added to evaluate what effect the choice of method for derivation of group ICs might have on the resulting FC spatial maps and time courses. This method used dual regression based on a single IC map selected from the group ICs derived with Infomax. We chose the dual-regression, single-map method because overall the fourth method produced spatial maps that most closely resembled the task-based GLM-derived spatial maps.

Each of the seven methods for deriving FC maps from group ICs was evaluated qualitatively through visual inspection of all spatial maps and time courses generated in this experiment and quantitatively according to the following criteria.

(1) *FC map correlation with task-related GLM spatial map from same subject > 0*: the same test as described for Experiment 2, but performed for each method across the 14 subjects.
(2) *FC map correlation with task-related GLM spatial map from same subject > FC map correlation with group IC map of interest (spatial prior)*: the same test as described for Experiment 2, but performed for each method across the 14 subjects, using the group IC map (from PICA for the first five methods, and Infomax for the other two methods).
(3) *FC map correlation with task-related GLM spatial map from same subject—a comparison of each method's mean correlation with that of the other six FC methods*: depending upon the results of a one-way repeated-measures ANOVA omnibus test of differences in mean correlations (*α* = 0.05), two-tailed, paired *t*-tests were performed to test for individual differences in means between methods. A Bonferroni correction for multiple comparisons with *α* = 0.05 was applied (significance at *P* < .0024 for each of the 21 method pairs compared).
(4) *Overlap of thresholded voxels from FC map with task-related GLM spatial map from same subject—a comparison of each method's overlap across subjects with the other six methods of deriving individual-subject FC maps*: the same test as described for Experiment 2, but performed for each method across the 14 subjects. A Bonferroni correction for multiple comparisons with *α* = 0.05 was applied (significance at *P* < .0024 for each of the 21 method pairs compared).
(5) *ROC PAUC from FC maps, using task-related GLM spatial maps as the standards of comparison*: for each subject and method evaluated, the partial area under the curve (PAUC) of the receiver operating characteristic (ROC) of the FC maps was calculated with FSL's fslmaths program. The task-related, GLM-derived map for each subject was used as the “true” standard of comparison. We chose for our PAUC the area between 0 and the largest false positive rate (LFPR) that we could accept, as in [73], because we did not wish to include area under portions of the ROC curve that would not be utilized in practice. We accepted the default suggested value for LFPR from the fslmaths program because false positive rates above this number (0.05) corresponded to the (unacceptable) situation where the number of false positives exceeded the total number of thresholded voxels in the reference maps (i.e., the number of thresholded voxels in the reference task-related GLM spatial maps in almost all cases was below 5% of the total number of voxels in the brain). We compared the PAUC values for each method to the other 6 methods, across the 14 runs, using the Wilcoxon signed rank test (two-tailed).

### 3.11. Experiment 4

We wanted to evaluate how the hybrid methods might perform using a considerably different number of ICs. To do so, we repeated the 5 evaluations from Experiment 3 for the 5 hybrid methods only, using seed group ICs generated with PICA that was set to produce only 5 ICs from the group data instead of 14 (the second of which was selected as the component of interest). We used the same number of seed voxels for the SV, FV, and MV methods as were used for Experiment 3. There is no consensus on how to select the optimal number of components [74], but some suggested methods for specifying dimensionality include the Laplace method, Bayesian information criterion (BIC), minimum description length (MDL), and Akaike's information criterion (AIC) [11, 12, 16]. We used each of these methods to estimate the number of components (through options in FSL MELODIC), resulting in dimensionalities of 11 (Laplace), 11 (AIC), 6 (MDL), and 5 (BIC). We chose the smallest dimensionality because we had already explored the highest number of components in using the Laplace option. The reason we ended up with 14 components instead of 11 is because MELODIC only rarely converged on a solution using a dimension of 11; so we accepted the MELODIC default alternative suggestion of 14.

### 3.12. Experiment 5

We repeated Experiment 4 using a much higher ICA dimensionality than was used in Experiments 3 and 4, to explore those effects that high dimensionality might have on hybrid method results. Statistical testing for Experiments 2–5 was performed with SPSS 12.0 for Windows.

## 4. Results

### 4.1. Experiment 1

ICA generated 17 components from Run 1. Upon visual inspection, one spatial map (Figure 2(b)) resembled the natural visual cortex component from GLM and one (Figure 2(c)) resembled the artificial component. The time course of the natural component (Figure 2(b)) resembled the time course of the voxel with the highest z-score from the task-related GLM time course (Figure 2(a)), and the square wave pattern of the artificial signal was evident in the time course of the artificial component (Figure 2(c)), despite the obvious presence of a considerable amount of noise. The natural component represented 2.46% of the total variance in the fMRI data (ranked 13th among components), and the artificial component represented 2.81% of the total variance (ranked 8th). Thresholded voxels of the artificial component spatial map corresponded to the voxels where signal had been added, with 100% sensitivity and specificity.

The ROI-based FC map (Figure 2(d)) derived using the voxel at MNI coordinates 21, −93, 9 as the seed contained thresholded voxels corresponding to both the natural and artificial components. Only 19 of the 984 voxels where artificial signal had been added were not represented in the map, corresponding to a sensitivity of 98.1%. The corresponding time course appeared to be somewhat similar to the time courses of both the natural and artificial components. It was not possible to discern the unique relationship that existed among the voxels involved with the natural component, or the unique relationship that existed among the voxels involved with the artificial component, because the spatial maps and time courses corresponding to the natural and artificial components were represented together in a single spatial map and time course.

The ROI-based FC map (Figure 2(e)) derived using the voxel with the highest z-score in the natural component spatial map showed robust activation in the occipital cortex without any activation in the voxels from the artificial component, except for the voxel at coordinates 21, −93, 9. The corresponding time course appeared very similar to the time course of both the voxel with the highest z-score from the task-related GLM (Figure 2(a)) and the natural component (Figure 2(b)). Similarly, the ROI-based FC map (Figure 2(f)) derived using the voxel with the highest z-score in the artificial component spatial map identified all of the voxels where artificial signal had been added (100% sensitivity), while only 10 of the whole brain's remaining 90,295 voxels were also thresholded (99.99% specificity). The corresponding time course was similar to the square wave pattern of the artificial signal. Thus, for these two cases the hybrid ICA-seed-based approaches resulted in spatial maps and time courses that were very similar to the seed IC spatial maps. It was possible to discern the two components as separate entities from these spatial maps and time courses.

### 4.2. Experiment 2

Figure 3(a) shows the spatial map and time course derived from task-related GLM for Run 3. Figures 3(b)–3(e) show the FC spatial maps and time courses derived from the first four hybrid methods using Run 3's IC13 as the seed source and fMRI data from Run 3. Figure 3(f) shows the FC spatial map and time course derived from the fifth hybrid method using all of Run 3's ICs as the seed source, with fMRI data from Run 3. Figure 3(g) shows IC13's spatial map and time course, and Figure 3(h) shows the spatial map that resulted from regressing the fMRI data against IC13's time course. Visual inspection revealed that the spatial maps and time courses in Figures 3(a)–3(h) were very similar to each other. Some thresholded voxels in the rTOF were found in all seven of these spatial maps.

**Figure 4:**
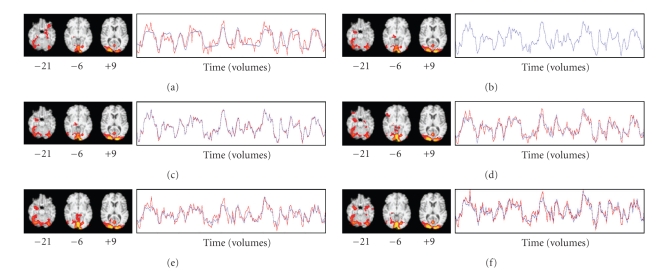
Spatial maps and time courses for Run 2: (a) task-related GLM; hybrid ICA-seed-based approaches, using the average time course from (b) a single voxel, (c) a few voxels, or (d) many voxels; and dual regression using seed spatial information from (e) an IC spatial map or (f) an entire set of IC spatial maps. Run 3 was the source of seed spatial information.

Figure 4(a) shows the spatial map and time course derived from task-related GLM for Run 2, which was the run with the second-highest z-score (11.5) in any voxel, derived from task-related GLM in response to viewing faces. Figures 4(b)–4(e) show the FC spatial maps and time courses derived from the first four hybrid methods using Run 3's IC13 as the seed source, with fMRI data from Run 2. Figure 4(f) shows the FC spatial map and time course derived from the fifth hybrid method using all of Run 3's ICs as the seed source, with fMRI data from Run 2. Visual inspection revealed that the spatial maps and time courses in Figures 4(a)– 4(f) were very similar to each other. Each spatial map showed an arm of a cluster extending rostrally from the right lateral occipital cortex into the rTOF, visible in the axial slice at *z* = −21 in Figures 4(a)–4(f).

**Figure 5:**
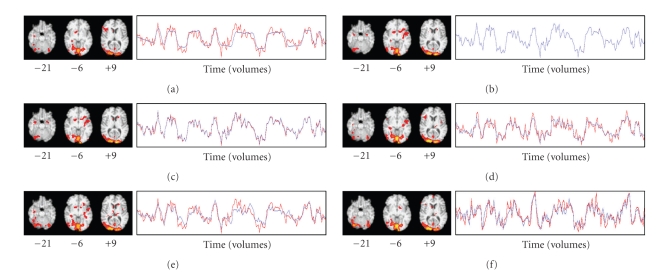
Spatial maps and time courses for Run 4: (a) task-related GLM; hybrid ICA-seed-based approaches, using the average time course from (b) a single voxel, (c) a few voxels, or (d) many voxels; and dual regression using seed spatial information from (e) an IC spatial map or (f) an entire set of IC spatial maps. Run 3 was the source of seed spatial information. No activation was detected in the rTOF in (b) and (c).

Figure 5(a) shows the spatial map and time course derived from task-related GLM for Run 4, which was the run whose highest z-score (10.4) in any voxel was the lowest among the six runs. Figures 5(b)–5(e) show the FC spatial maps and time courses derived from the first four hybrid methods using Run 3's IC13 as the seed source, with fMRI data from Run 4. Figure 5(f) shows the FC spatial map and time course derived from the fifth hybrid method using all of Run 3's ICs as the seed source, with fMRI data from Run 4. Visual inspection revealed that the spatial maps and time courses in Figures 5(a)–5(f) were similar to each other. However, no thresholded voxels were found in the rTOF for the first two hybrid methods (the single-voxel and few-voxel hybrid methods). Other than these two cases and the task-related GLM map for Run 5, at least one thresholded voxel was found in the rTOF in all task-related and FC spatial maps.

Results from the quantitative analyses were as follows.

**Criterion**

All mean correlations (“Same” column in Table 1) were significantly greater than zero (*P* < .001). In addition, the range of mean correlations (0.71–0.79) compared favorably with the range of correlations of the task-related GLM maps with each other (0.13–0.45).

**Criterion**

For each hybrid method, the mean correlation between hybrid FC maps and the task-related GLM spatial map from the same run was greater than the mean correlation between hybrid FC maps and the task-related GLM spatial map from the seed run, with *P* ≤ .001 (Table 1).

**Criterion**

The omnibus test of differences in mean correlations (“Same” column, Table 1) was nonsignificant (*F* = 2.1, *P* = .13).

**Criterion**

The threshold-adjusted overlap percentages were comparable for each hybrid method (Table 2). The MV approach had better coverage of its task-related GLM maps for all five target runs than the SV, FV, and DRA approaches (*P* = .043), but this result was nonsignificant after correction for multiple comparisons.

**Criterion**

No statistically significant differences were found between the five target-run, task-related GLM maps and the five target-run FC maps for each of the five hybrid methods: 4/5 of the task-related GLM maps contained at least one thresholded voxel in the rTOF compared with 4/5 of the FC spatial maps for the single-voxel and few-voxel hybrid methods and 5/5 of the FC spatial maps for the many-voxel hybrid method and for both dual regression hybrid methods.

### 4.3. Experiment 3

Results from the quantitative analyses were as follows.

**Criterion**

All mean correlations (“Task-Related” column in Table 3) were significantly greater than zero (*P* < .001).

**Criterion**

Except for DRA (nonsignificant), FC maps generated with the hybrid methods were significantly more correlated with the corresponding task-related GLM maps than with the group IC seed sources (Table 3). In contrast, FC maps generated with BR-I were significantly less correlated with the corresponding task-related GLM maps than with the Infomax group IC of interest (IC14).

**Criterion**

The omnibus test showed that the mean correlations (“Task-Related” column in Table 3) differed significantly across methods (*F* = 80.3, *P* < .001). Post hoc, paired *t*-test analyses with Bonferroni correction revealed that the mean correlation for BR-I was significantly lower than the mean correlations for the other six methods; the mean correlation for DRA was significantly lower than the mean correlations for the remaining five methods; and the mean correlation for DRS-I was lower than the mean correlation for DRS.

**Criterion**

The threshold-adjusted overlap percentages were significantly lower for BR-I than those for the other six methods and significantly greater for DRS than for DRA and DRS-I (Table 4).

**Criterion**

The ROC PAUCs were significantly lower for BR-I than for the other six methods; significantly greater for DRS than those for DRA and DRS-I; and significantly greater for MV than for DRS-I (data not shown).

**Qualitative Analyses**

We examined the data qualitatively to understand why these results were better (more like the task-related GLM results) for some of the subjects than for others. An examination of the ROC curves and PAUCs by subject (across methods) revealed that 13 of the subjects showed highly similar patterns and ranged in mean adjusted PAUC (scaled to a maximum possible area of 1) from 0.52 to 0.79. In contrast, one of the subjects (Subject 8) had a mean adjusted PAUC of 0.24, and the data for this subject appeared to deviate in many respects from the others. Therefore, we selected Subject 8's data for further study and compared this data to that from Subject 1 (highest mean PAUC). The ROC curves for Subject 1 and Subject 8 are shown in Figures 7 and 8.

**Figure 6:**
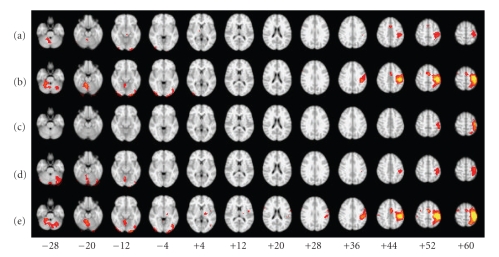
Group spatial maps derived from (a) task-related GLM; (b) group ICA, PICA, *N* = 14; (c) group ICA, Infomax, *N* = 14; (d) group ICA, PICA, *N* = 5; and (e) group ICA, PICA, *N* = 30. *N* is the number of ICs generated.

**Figure 7:**
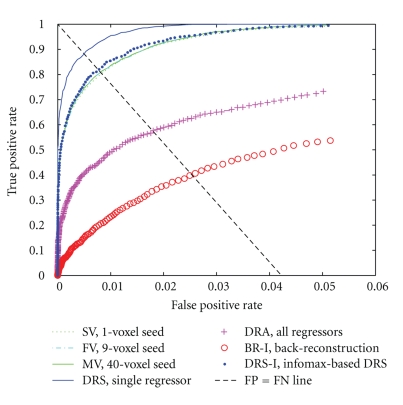
ROC curves for Subject 1, on the interval from 0 to 0.05. Subject 1's task-related, GLM-derived spatial map was used as the “true” standard of comparison. The points where the number of false positives (FPs) was equal to the number of false negatives (FNs) fall along the FP = FN line shown above. This helps to explain why the results of the ROC PAUC analyses (Experiments 3–5, Criterion 5) were so similar to the results of the threshold-adjusted overlap analyses (Experiments 3–5, Criterion 4).

**Figure 8:**
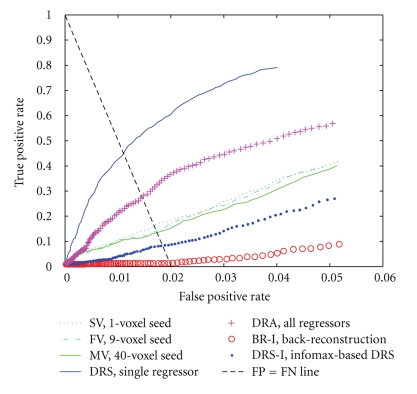
ROC curves for Subject 8, on the interval from 0 to 0.05. Subject 8's task-related, GLM-derived spatial map was used as the “true” standard of comparison.

**Figure 9:**
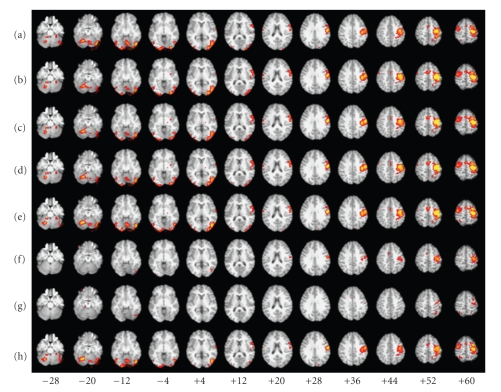
Spatial maps derived from Subject 1's fMRI data using (a) task-related GLM; (b) single-voxel hybrid method (SV); (c) few-voxel hybrid method (FV); (d) many-voxel hybrid method (MV); (e) dual regression, single IC hybrid method (DRS); (f) dual regression, all IC hybrid method (DRA); (g) back-reconstruction with Infomax (BR-I); and (h) dual regression, single IC hybrid method, Infomax group IC seed source (DRS-I).

**Figure 10:**
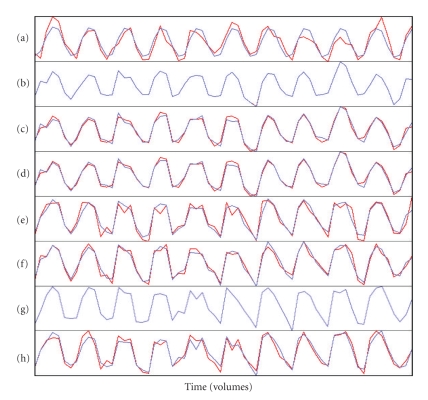
Time courses derived from Subject 1's fMRI data using (a) task-related GLM; (b) single-voxel hybrid method (SV); (c) few-voxel hybrid method (FV); (d) many-voxel hybrid method (MV); (e) dual regression, single IC hybrid method (DRS); (f) dual regression, all IC hybrid method (DRA); (g) back-reconstruction with Infomax (BR-I); and (h) dual regression, single IC hybrid method, Infomax group IC seed source (DRS-I). Blue lines: time courses from linear model. Red lines: fMRI data where deviating from model, at voxel with best fit to model.

**Figure 11:**
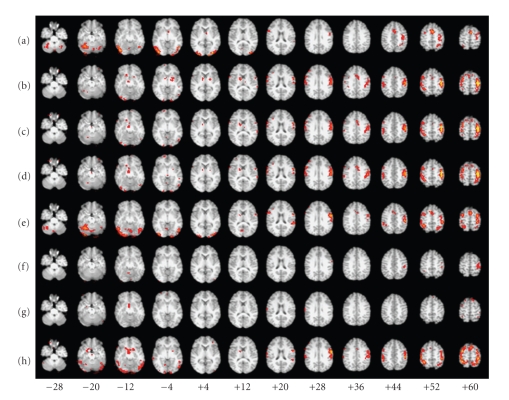
Spatial maps derived from Subject 8's fMRI data using (a) task-related GLM; (b) single-voxel hybrid method (SV); (c) few-voxel hybrid method (FV); (d) many-voxel hybrid method (MV); (e) dual regression, single IC hybrid method (DRS); (f) dual regression, all IC hybrid method (DRA); (g) back-reconstruction with Infomax (BR-I); and (h) dual regression, single IC hybrid method, Infomax group IC seed source (DRS-I).

**Figure 12:**
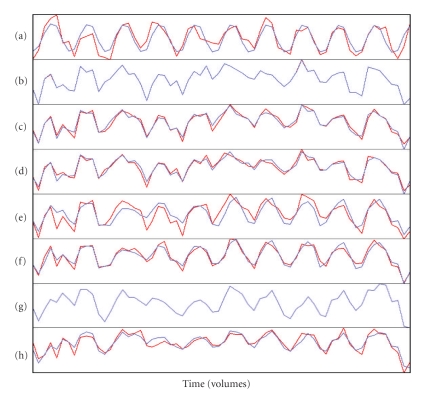
Time courses derived from Subject 8's fMRI data using (a) task-related GLM; (b) single-voxel hybrid method (SV); (c) few-voxel hybrid method (FV); (d) many-voxel hybrid method (MV); (e) dual regression, single IC hybrid method (DRS); (f) dual regression, all IC hybrid method (DRA); (g) back-reconstruction with Infomax (BR-I); and (h) dual regression, single IC hybrid method, Infomax group IC seed source (DRS-I).

For Subject 1, the FC maps derived with each method showed a fairly robust “activation” in sensorimotor (primarily left) and visual cortices (Figures 9(b)– 9(h)), and the corresponding time courses showed the expected cyclical time course of brain activity in response to the 10 repeated 24-second blocks of the experimental paradigm (Figures 10(b)–10(h)). These spatial maps and time courses resembled Subject 1's task-related GLM spatial map (Figure 9(a)) and time course (Figure 10(a)). For Subject 8 we also saw “activation” in sensorimotor and visual cortices (Figures 11(b)–11(h)) but the regions of “activation” were smaller and not as clearly lateralized to the left as for Subject 1. The activation pattern for Subject 8's task-related GLM map (Figure 11(a)) was smaller and missing posterior portions of the sensorimotor cortex that were visible for Subject 1. Subject 8's FC maps bore some resemblance to the task-related GLM map, except in the case of BR-I, which appeared not to bear any resemblance (spatial correlation 0.10 and threshold-adjusted “activation” overlap 0.3%); however, the spatial correlation of BR-I's FC map (Figure 11(g)) with Infomax IC14 (Figure 6(c)) was 0.59. The FC time courses for Subject 8 (Figures 12(b)–12(h)) resembled Subject 8's task-related GLM time course (*a priori* time course in blue, Figure 12(a)) with 10 equidistant peaks roughly discernable, but these were not as close to the task-related GLM time course as for Subject 1.

We also examined the data to understand why the results were better for some of the FC methods than for others. The DRS and DRA methods differed greatly in how well their FC maps matched the corresponding task-related GLM maps, yet the only difference between the two methods was that 13 nuisance regressors were added to the linear model for DRA. Some of those regressors had a very similar time course to that of PICA IC2, and all but one of the 14 ICs had a peak in its time course power spectrum at 0.042 Hz (the frequency corresponding to the block period of the experiment), with most of those peaks being the highest peak for the component. Also, most of the IC spatial maps included some subthreshold or barely suprathreshold “activation” in the left sensorimotor brain region. Inspection of the Infomax ICs revealed similar relationships between Infomax IC14 and the other Infomax group ICs.

### 4.4. Experiment 4

Results from the quantitative analyses were as follows.

**Criterion**

All mean correlations (“Task-Related” column in Table 5) were significantly greater than zero (*P* < .001).

**Criterion**

Except for the SV and DRA methods, FC maps generated with the hybrid methods were significantly more correlated with the corresponding task-related GLM maps than with the group IC seed sources (Table 5).

**Criterion**

The omnibus test showed that the mean correlations (“Task-Related” column in Table 5) differed significantly across methods (*F* = 19.1, *P* < .001). Posthoc, paired *t*-test analyses with Bonferroni correction revealed that the mean correlations for FV, MV, and DRS were significantly greater than those for SV and DRA.

**Criterion**

The threshold-adjusted overlap percentages were significantly lower for SV than those for FV, MV, and DRS; and significantly lower for DRA than those for MV (Table 6).

**Criterion**

The ROC PAUCs were significantly lower for SV than those for FV and MV and significantly lower for DRA than those for MV (data not shown).

**Qualitative Analyses**

We examined the data qualitatively to understand why the results in Experiment 4 were better for the FV, MV, and DRS methods than those for SV, which had not been the case in Experiment 3. Examination of the PICA group ICs from Experiments 3 and 4 showed that the spatial maps and time courses for the group ICs of interest (IC2, in both cases) were highly similar (*r* = 0.70) for both experiments, but while Experiment 3's IC2 had the highest z-scores in a large contiguous region in the left sensorimotor cortex, Experiment 4's IC2 had its highest z-scores distributed to voxels in the same region of the sensorimotor cortex and to smaller regions in caudal and ventral portions of the left visual cortex, in peripheral brain regions. For this reason, in Experiment 4 the SV method chose a seed voxel in the visual cortex rather than in the sensorimotor cortex. In contrast, the FV and MV methods chose seed voxels that were split between the visual and sensorimotor cortices, with most voxels in the latter.

### 4.5. Experiment 5

Results from the quantitative analyses were as follows.

**Criterion**

All mean correlations (“Task-Related” column in Table 7) were significantly greater than zero (*P* < .001).

**Criterion**

FC maps generated with the DRA method were significantly less correlated with the corresponding task-related GLM maps than with the group IC seed sources (Table 7). FC maps generated with the other four hybrid methods were significantly more correlated with the corresponding task-related GLM maps than with the group IC seed sources.

**Criterion**

The omnibus test showed that the mean correlations (“Task-Related” column in Table 7) differed significantly across methods (*F* = 62.4, *P* < .001). Posthoc, paired *t*-test analyses with Bonferroni correction revealed that the mean correlations for SV, FV, MV, and DRS were significantly greater than those for DRA and the mean correlations for DRS were significantly greater than for those FV (*P* = .004, uncorrected) and MV (*P* = .004), but not SV (*P* = .006).

**Criterion**

The threshold-adjusted overlap percentages were significantly lower for DRA than those for SV, FV, MV, and DRS (Table 8).

**Criterion**

The ROC PAUCs were significantly lower for DRA than those for SV, FV, MV, and DRS (data not shown). In Experiments 3–5, the *P*-values for the comparisons using ROC PAUC were very similar to those obtained from using threshold-adjusted overlap. Differences in reported statistical significance only occurred for cases where *P*-values fell close to the threshold for statistical significance.

**Qualitative Analyses**

We examined the data qualitatively to understand why the results in Experiment 5 were similar to those from Experiment 3, but not Experiment 4. The group IC spatial maps for Experiments 3 and 5 were very similar in appearance (Figures 6(b) and 6(e)) and highly correlated (0.87). This similarity apparently resulted in very similar locations for the seed voxels for the SV, FV, and MV methods. The main difference in the locations was that the seed voxels for Experiment 5 appeared to be shifted posteriorly by one or two voxels compared with those from Experiment 3.

## 5. Discussion

The main purpose of this study was to perform a small-scale comparison of several hybrid ICA-seed-based methods of FC assessment to determine which, if any, of these methods might merit further evaluation in larger-scale studies, to determine their potential usefulness compared with other, standard methods of assessing FC. All five hybrid methods performed well in each of four experiments (Experiments 2–5), producing spatial maps that were consistently significantly and highly spatially correlated (>0.7 for FV, MV, and DRS; >0.5 for SV; and >0.4 for DRA) with the corresponding task-related GLM maps. These correlations were significantly higher than the corresponding correlations with the seed sources, except for the SV method in Experiment 4 and the DRA method in Experiments 3–5, indicating that the spatial maps produced were a reflection of the individual subject/session's data rather than possibly being unduly influenced by the seed source. Also, in Experiment 2 the hybrid methods were not inferior to task-related GLM in their capacity to detect “activation” in the rTOF from fMRI data collected during multiple runs where images of faces were viewed. These results suggest that, at least for some applications, it is possible to derive FC maps with GLM, based on seeds from an exploratory ICA, resulting in FC maps that resemble the seed IC(s); yet the FC maps are unique to the fMRI data being analyzed. This study also demonstrates that the ICA and FC analyses need not be derived from the same data.

The main motivation for exploration of hybrid ICA-seed-based FC methods is the possibility that such methods will allow production of FC spatial maps and time courses without the limitations of each method performed separately. Hybrid ICA-seed-based FC methods utilize the exploratory power of ICA in the generation of one or more seed ICs, while providing a rationale for choice of seed. Such a rationale may be helpful in cases where a choice of seed based on theoretical considerations might seem arbitrary and chosen from a large number of possibilities, although some theoretical considerations might still be needed to select the seed IC or IC of interest. Hybrid ICA-seed-based FC methods might have the discerning power of ICA to provide accurate information about special relationships that exist among subpopulations of voxels in FC maps, as demonstrated in Experiment 1. This capacity can be particularly helpful in cases where GLM seed-based FC maps would otherwise be generated from seed voxels where multiple components overlap. Finally, hybrid ICA-seed-based FC methods allow circumvention of statistical complications arising from reproducibility issues associated with group comparison of spatial maps produced by ICA, because the last step of such methods involves GLM rather than ICA. For the SV, FV, and MV hybrid methods this last step is a standard ROI, seed-based assessment of FC, where ICA has provided the choice of seed ROI. The DRS hybrid method is not much different conceptually, because rather than averaging the voxel time courses within an ROI, a “weighted average” is performed through least-square linear regression, which heavily weights the values of the derived time course toward those of the voxels with highest z-scores in the seed IC.

Previous investigations into the potential benefits of hybrid ICA-seed-based FC approaches have not been reported in the literature, to the authors' knowledge. However, a number of approaches designed to utilize the exploratory power of ICA while addressing reproducibility issues involved with group comparison of ICA-derived spatial maps have been described [12, 26, 31, 37, 38, 75, 76]. Some of these require prior temporal information [37, 38, 76] and are therefore not suitable when such information is unavailable, as in resting-state studies. Among methods suitable for resting state studies, one of the first proposed methods is BR-I [12], compared in this study. It makes intuitive sense that restricting ICs for all subjects to be based on a single group ICA would limit the intersubject variability for spatial maps corresponding to each component, but it remains unclear whether this results in truly homologous groups of ICs to be compared (i.e., ICs based on signals having exactly the same neural origins) across subjects. Another problem with the “back-reconstruction” approach is reproducibility across studies: the stochastic nature of ICA means that group ICs derived in one study might not exactly match the ICs from another study with different participants. For example, even consistently reproducible ICs such as the “default mode” component [8, 74] can sometimes be split into two components [77, 78]. These issues are also potentially problematic with the “multisubject ICA and dual regression” approach [31].

Seed-based approaches can address this last issue by utilizing fixed spatial priors to facilitate reproducibility across studies. Such maps can be derived from ICA or task-related GLM or artificially constructed based on theoretical considerations. Instead of deriving seed spatial maps from all subjects being compared, it may be advantageous in some cases to derive seed spatial maps from an outside reference group of subjects. For example, it might be desirable to compare FC results from a current study with those of a previous study by deriving FC from exactly the same seed maps that were used in the previous study, particularly if the previous study involves a much larger sample size. When healthy and diseased samples of subjects are compared, it might be advantageous in some cases to use seed maps derived from exclusively healthy or exclusively diseased subjects, rather than seed maps derived from a mixed sample. Finally, in cases where a group IC of interest appears (by chance) to be fragmented into multiple ICs, rather than attempting to piece together the fragments or to use them individually as seeds for dual-regression-based FC analysis, it might be better to choose seeds from a previously derived IC or set of ICs that are deemed prototypical of the spatial patterns of interest.

Another approach that utilizes fixed spatial priors is spatial semiblind spatial ICA (SSS-ICA) [26]. SSS-ICA uses predetermined spatial maps to guide ICA toward more correct solutions and was demonstrated to yield higher signal-to-noise ratios than Infomax ICA [57] and ICA-with-reference algorithms (ICA-R) [79], particularly with an accurate spatial prior under low-noise conditions [26]. It would be useful to know to what extent SSS-ICA might unduly influence the resulting spatial maps to resemble the spatial priors. SSS-ICA was reported not to be influenced by an incorrect spatial prior in the absence of noise [26], but further exploration of this question would be helpful. Another reported ICA method involving spatial priors that can be used without *a priori* temporal information is fixed-average spatial ICA (FAS-ICA) [75], which is similar to hybrid ICA-seed-based FC methods because it involves regression of fMRI data against spatial maps generated with ICA. With FAS-ICA, the spatial maps used as seeds are generated by averaging the IC spatial maps derived from performing BR-I on multiple fMRI runs for the same subject under one condition; then the fMRI data from another condition is regressed against the complete set of averaged IC spatial maps (averaged across homologous ICs from different runs) to generate corresponding time courses. If one were to add, as a final step to FAS-ICA, regression of the individual-subject fMRI data against those time courses to generate corresponding spatial maps, this would be equivalent to the DRA approach described here (using an averaged set of IC spatial maps derived with BR-I as the seed spatial maps).

The hybrid ICA-seed-based FC methods FV, MV, and DRS performed the best overall in Experiments 2–5. The high correlations, overlaps, and ROC PAUCs with the hypothesis-driven, reference task-related GLM spatial maps serve as confirmation that those hybrid methods produced meaningful and valid results that reflected brain neural activity. However, the relatively “poor” results obtained in some cases by the SV and DRA methods and in every case by the BR-I method do not serve as a disconfirmation of the validity of those methods. For example, it is possible that the results from both BR-I and task-related GLM were highly valid, accurately reflecting brain activity during the experiment described in [49]. These methods may have reflected somewhat separate elements of the total brain activity that was occurring during the experiment. From the qualitative analyses for Experiment 3 it appeared as if the spatial and temporal properties of the task-related GLM were split among several Infomax group IC's, and the same was true for the PICA group ICs, which helps to explain why the DRA spatial maps were not as closely related to the task-related GLM maps as were the spatial maps of the other hybrid methods. Although these findings do not indicate that the DRA and BR-I methods are inferior to the first four hybrid methods, they do not provide support for their use either; and a reason to be discouraged from using these two methods and any method that directly generates with ICA the spatial maps to be used for groupwise comparisons is the risk of not making a valid comparison across homologous-looking components, due to a seemingly random (because we do not fully understand this process) distribution of fMRI variance into the various components. Until we know more about the conditions under which ICA will split variance from one component into separate, temporally related components, it may be safer to use seed-based or hybrid seed-based methods to derive FC maps used for group inferences.

The hybrid (and seed-based) FC methods are also affected by factors that can decrease the validity of group inferences, but these are issues that are more easily understood and addressed and that affect nearly all types of fMRI data analyses. For example, factors that might have contributed to the somewhat different appearance of Subject 8's task-related GLM and FC maps compared with the other subjects include differing activities during the experiment, differing intensities of responses, differing brain hemodynamic responses or response times, differing brain anatomy, differing localization of areas of brain function, and differing specialization of functions used to perform the same task [80]. Although the latter four factors can be problematic and invalidate or reduce the sensitivity of results of fMRI analyses, these are relatively easier to understand and are amenable to being addressed with experimental measures. For example, a brain localizer scan or improved methods of coregistration of different brains to each other and to a standard brain can help to reduce the effect of such factors [81, 82].

Which among the hybrid ICA-seed-based FC methods SV, FV, MV, and DRS is best is unclear and may depend upon the FC application in question. The SV method has the advantage of being the easiest to report and reproduce, because the only information needed is the coordinates of a single voxel. The SV method should in most cases be comparable to the FV and MV methods (as in Experiments 2, 3, and 5), because after spatial smoothing, the voxel's time course and variance will become more similar to its neighbors. However, Experiment 4 underscored a potential weakness of the SV method. In order for the SV method to work well, the single voxel should preferably be placed in the middle of a large area of activation, typical for the type of activity being studied. If the voxel is placed in the periphery, on the border between brain areas with widely differing functions, or in a brain area not well representative of the activity being studied, then use of the FC maps that result may not be suitable for groupwise comparison due to heterogeneity of the spatial maps and time courses that may result. Thus, if we use the SV method, it is important to inspect the data to determine the appropriateness of the location of the single voxel. If automated methods are used it may be safer to use the DRS method, which includes information from the entire seed spatial map and which performed the best overall in Experiments 2–5.

We mention four study limitations. First, different thresholding levels and scaling used for spatial maps derived with PICA and Infomax may have caused the PICA-derived maps to appear more robust than the Infomax-derived maps, even though we were using a relatively conservative thresholding level for PICA-derived maps. However, the primary measures we used for statistical testing (spatial correlation, threshold-adjusted overlap, and PAUC) were not influenced by spatial map scaling and threshold cutoffs. Second, the dimensionalities used in Experiments 3 and 4 were both lower than those typically reported in the literature for group ICA, where the number of components has often been set manually to a round number such as 20 or 30 [39, 58, 75, 83, 84], and when estimated automatically has typically been greater than 15 [6, 85]. One possible explanation for the low estimates in our study could be that they accurately reflected a relatively low amount of structured noise in the fMRI data [29], but the most appropriate choice of dimensionality is not clear and therefore remains somewhat arbitrary. However, the hybrid methods as a group performed well for the wide range of dimensionalities tested in this study. Third, although we have demonstrated that the hybrid methods can work on task-related data, we have not demonstrated that they can work on resting-state data, for which such hybrid methods are primarily intended. We chose experimental paradigms associated with activation of visual and sensorimotor brain regions because such paradigms generally result in strong and predictable fMRI activation patterns, which facilitated confirmation of the validity of the FC spatial maps and time courses derived with hybrid methods. Also, the use of such paradigms is commonly reported in the literature for testing of FC- and ICA-related methods and to compare FC- and ICA-related spatial maps to those derived based on hypothesis testing with *a priori* time courses related to the experimental paradigm [12, 26, 35, 37, 75, 86]. We believe that the comparisons here can be extrapolated to resting-state studies because a number of studies have demonstrated the parallels between resting-state activity and task-related activity. For example, group ICA studies have demonstrated that 8–10 group ICs (depending upon the group ICA method used) can consistently be found across subjects, resting-state sessions, and within sessions (with bootstrapping) with BOLD signal changes of up to 3%, comparable to those found for task-related studies [6, 87, 88]. In [23], single-subject ICs exhibited an extremely high degree of consistency in spatial, temporal, and frequency parameters within and between subjects. Reproducibility across subjects and sessions was also found for resting-state, ROI-based FC [89], and although such ROI-based FC was found to frequently correspond to structural connections as measured with diffusion spectrum imaging tractography, some FC was not explained by direct structural connections [90]. In resting state, the activity of such functionally connected regions is frequently correlated with functionally related areas [35, 78] and such activity levels are modulated by task activity [85], resulting in increased FC in functionally relevant areas being exercised by the experimental paradigm [10]. Fourth, another possible limitation in the conclusions that may be drawn from this study concerns the question of whether it is appropriate to measure task-related brain activity using seed-based FC measures, which are fundamentally different than measures based on *a priori* time courses, because the time courses of the former are based upon the temporal variations at one or more brain regions, while the time courses of the latter are based upon the temporal variations that are expected in response to an experimental paradigm. The differences between these approaches should not differ by much provided that the seed location corresponds to a brain location whose activity is sufficiently synchronous with the hemodynamic response to the experimental paradigm. As long as the chosen location is functionally related to the experimental task, this should not be a problem (unlike the case for the SV method in Experiment 4).

In conclusion, our experiments demonstrate that at least four of the hybrid ICA-seed-based FC methods tested (SV, FV, MV, and DRS) can produce spatial maps and time courses that correspond closely to what would be expected from knowledge of the experimental paradigm in play during fMRI data collection and from examination of spatial maps and time courses generated with task-related GLM, based on hypothesis testing with *a priori* time courses. We hope that further empirical and theoretical studies will help to elucidate what methods for data-driven extraction of components (spatial maps and time courses) are most likely to improve the validity of groupwise comparisons. Our findings suggest that one or more hybrid ICA-seed-based methods should be included in such studies. Until we know more about factors that govern the seemingly stochastic nature of how ICA parses fMRI variance into temporally related components, it may be safer to use hybrid ICA-seed-based FC methods to generate ICA-related spatial maps and time courses used for groupwise comparison. The use of such methods effectively channels exploratory information from ICA into the realm of seed-based FC, which for years has been widely accepted, understood, and implemented by the imaging community [1, 2, 91, 92].

## Figures and Tables

**Table 1:** Experiment 2: mean correlations of hybrid ICA-seed-based maps with maps from task-related GLM, over the five target (nonseed) fMRI data sets. “Same” indicates mean correlation of hybrid method FC maps with task-related GLM derived from the same fMRI dataset. “Seed” indicates mean correlation of hybrid method FC maps with task-related GLM map from Run 3's fMRI dataset, the seed source.

Source of time course regressor(s)	Mean correlation with task-related map (95% Confidence Interval)		Same = Seed Null Hypothesis Test		
	Same	Seed	*t*	df	*P*
Single voxel (SV)	0.71 (0.63–0.78)	0.23 (0.15–0.31)	11.3	4	<.001
Average over 5 voxels (FV)	0.71 (0.64–0.77)	0.23 (0.15–0.30)	15.2	4	<.001
Average over 118 voxels (MV)	0.79 (0.75–0.83)	0.33 (0.28–0.38)	19.1	4	<.001
Regression against 1 spatial map (DRS)	0.72 (0.66–0.77)	0.28 (0.21–0.35)	11.5	4	<.001
Regression against all spatial maps (DRA)	0.74 (0.69–0.79)	0.40 (0.39–0.41)	8.7	4	.001

**Table 2:** Experiment 2: mean percentages of thresholded task-related GLM maps that are covered by thresholded FC maps generated with each hybrid ICA-seed-based method. The right-most column gives percentages after adjusting the z-score used to threshold the FC maps up or down so that number of false positives equals number of false negatives.

Source of time course regressor(s)	Percent of Task-Related Map Covered (95% Confidence Interval)	
	Before Threshold Adjustment	After Threshold Adjustment
Single voxel	94 (92–97)	78 (72–83)
Average over 5 voxels	94 (92–97)	78 (73–83)
Average over 118 voxels	95 (93–98)	87 (84–90)
Regression against 1 spatial map	95 (93–98)	84 (81–87)
Regression against all spatial maps	96 (94–97)	83 (80–85)

**Table 3:** Experiment 3: mean correlations of subject-specific FC spatial maps with (1) the task-related GLM map for the same subject (“Task-Related” column) and (2) the group IC of interest (“Group IC” column). Results of a paired *t*-test comparison of the mean correlations are shown in the rightmost columns. Higher correlations with the task-related maps than with the group IC of interest are an indication that the results reflect the individual-subject data without being overly biased by spatial features of the group IC. The group ICs were generated with PICA or Infomax. These results were based on group ICAs that produced 14 components.

Method of Generating Maps	Group ICA Source	Mean Correlation With Subject-Specific Map (95% Confidence Interval)		Same = Seed Null Hypothesis Test		
		Task-Related	Group IC	*t*	df	*P*
Single-voxel seed	PICA	0.84 (0.77–0.89)	0.52 (0.48–0.55)	7.7	13	<.001
9-voxel ROI seed	PICA	0.85 (0.79–0.89)	0.53 (0.49–0.57)	8.2	13	<.001
40-voxel ROI seed	PICA	0.86 (0.80–0.90)	0.54 (0.49–0.58)	8.9	13	<.001
Dual regression, 1 spatial map	PICA	0.88 (0.84–0.91)	0.55 (0.52–0.58)	10.0	13	<.001
Dual regression, all spatial maps	PICA	0.53 (0.47–0.59)	0.59 (0.56–0.61)	−2.3	13	.04
Back-reconstruction	Infomax	0.30 (0.22–0.38)	0.62 (0.58–0.66)	−10.4	13	<.001
Dual regression, 1 spatial map	Infomax	0.81 (0.73–0.87)	0.43 (0.40–0.46)	6.6	13	<.001

**Table 4:** Experiment 3: mean percentages of thresholded task-related GLM maps that are covered by the thresholded subject-specific FC maps generated with each method below. The right-most column gives percentages after adjusting the z-score used to threshold the FC maps so that number of false positives equals number of false negatives. For the BR-I method, adjusting the threshold helped to compensate for what might have been an overly conservative thresholding, but the results for this method were still significantly lower those than for the other six methods used.

Method of Generating Maps	Group ICA Source	Percent of Task-Related Map Covered (95% Confidence Interval)	
		Before Threshold Adjustment	After Threshold Adjustment
Single-voxel seed (SV)	PICA	57 (46–69)	63 (54–73)
9-voxel ROI seed (FV)	PICA	61 (51–71)	64 (55–74)
40-voxel ROI seed (MV)	PICA	65 (55–75)	66 (56–75)
Dual regression, 1 spatial map (DRS)	PICA	78 (70–85)	71 (64–78)
Dual regression, all spatial maps (DRA)	PICA	28 (20–36)	50 (43–57)
Back-reconstruction (BR-I)	Infomax	9 (6–12)	29 (21–36)
Dual regression, 1 spatial map (DRS-I)	Infomax	59 (47–72)	56 (45–68)

**Table 5:** Experiment 4: mean correlations of subject-specific FC spatial maps with (1) the task-related GLM map for the same subject (“Task-Related” column) and (2) the group IC of interest (“Group IC” column). Results of a paired *t*-test comparison of the mean correlations are shown in the rightmost columns. These results were based on a group ICA that produced 5 components.

Method of Generating Maps	Group ICA Source	Mean Correlation With Subject-Specific Map (95% Confidence Interval)		Same = Seed Null Hypothesis Test		
		Task-Related	Group ICA	*t*	df	*P*
Single-voxel seed (SV)	PICA	0.57 (0.45–0.68)	0.47 (0.43–0.51)	2.0	13	.06
9-voxel ROI seed (FV)	PICA	0.81 (0.73–0.86)	0.53 (0.49–0.57)	5.8	13	<.001
40-voxel ROI seed (MV)	PICA	0.83 (0.77–0.87)	0.56 (0.52–0.60)	7.0	13	<.001
Dual regression, 1 spatial map (DRS)	PICA	0.77 (0.67–0.83)	0.59 (0.57–0.61)	3.7	13	.002
Dual regression, all spatial maps (DRA)	PICA	0.57 (0.46–0.65)	0.62 (0.59–0.64)	−1.2	13	.24

**Table 6:** Experiment 4: mean percentages of thresholded task-related GLM maps that are covered by the thresholded subject-specific FC maps generated with each hybrid method below. The right-most column gives percentages after adjusting the z-score used to threshold the FC maps so that number of false positives equals number of false negatives. In contrast with Experiment 3, the results for the SV method were significantly lower than those for the FV, MV, and DRS methods. The difference between Experiments 3 and 4 was that Experiment 4 was based on a group ICA that produced 5 components rather than 14. As explained in the text, the differences in results between these two experiments can be accounted for by subtle differences between the seed-source ICs, which led to markedly different brain locations for the single-voxel seed.

Method of Generating Maps	Group ICA Source	Percent of Task-Related Map Covered (95% Confidence Interval)	
		Before Threshold Adjustment	After Threshold Adjustment
Single-voxel seed (SV)	PICA	15 (6–24)	25 (15–35)
9-voxel ROI seed (FV)	PICA	47 (35–59)	54 (43–65)
40-voxel ROI seed (MV)	PICA	57 (45–69)	57 (46–68)
Dual regression, 1 spatial map (DRS)	PICA	46 (33–60)	44 (32–56)
Dual regression, all spatial maps (DRA)	PICA	33 (22–44)	40 (29–50)

**Table 7:** Experiment 5: mean correlations of subject-specific FC spatial maps with (1) the task-related GLM map for the same subject (“Task-Related” column) and (2) the group IC of interest (“Group IC” column). Results of a paired *t*-test comparison of the mean correlations are shown in the rightmost columns. These results were based on a group ICA that produced 30 components.

Method of Generating Maps	Group ICA Source	Mean Correlation WithSubject-Specific Map(95% Confidence Interval)		Same = SeedNull HypothesisTest		
		Task-Related	Group ICA	*t*	df	*P*
Single-voxel seed (SV)	PICA	0.82 (0.75–0.88)	0.48 (0.44–0.52)	7.5	13	<.001
9-voxel ROI seed (FV)	PICA	0.84 (0.77–0.89)	0.49 (0.45–0.53)	7.5	13	<.001
40-voxel ROI seed (MV)	PICA	0.85 (0.79–0.89)	0.50 (0.45–0.54)	8.3	13	<.001
Dual regression, 1 spatial map (DRS)	PICA	0.88 (0.84–0.92)	0.51 (0.48–0.54)	9.8	13	<.001
Dual regression, all spatial maps (DRA)	PICA	0.43 (0.36–0.49)	0.52 (0.48–0.55)	−4.0	13	.002

**Table 8:** Experiment 5: mean percentages of thresholded task-related GLM maps that are covered by the thresholded subject-specific FC maps generated with each hybrid method below. The right-most column gives percentages after adjusting the z-score used to threshold the FC maps so that number of false positives equals number of false negatives. The results are similar to those from Experiment 3, with DRA covering less of the task-related maps than the SV, FV, MV, and DRS methods.

Method of Generating Maps	Group ICA Source	Percent of Task-Related Map Covered (95% Confidence Interval)	
		Before Threshold Adjustment	After Threshold Adjustment
Single-voxel seed (SV)	PICA	54 (44–63)	62 (52–71)
9-voxel ROI seed (FV)	PICA	58 (48–68)	63 (53–72)
40-voxel ROI seed (MV)	PICA	62 (52–72)	64 (54–74)
Dual regression, 1 spatial map (DRS)	PICA	76 (68–85)	69 (61–78)
Dual regression, all spatial maps (DRA)	PICA	12 (7–16)	43 (36–50)
